# Commentary: Research trends and hotspots of post-stroke dysphagia rehabilitation: a bibliometric study and visualization analysis

**DOI:** 10.3389/fnins.2024.1357272

**Published:** 2024-03-07

**Authors:** Maomao Huang, Chunyu Zhang, Zige Liu, Jianxiong Wang

**Affiliations:** ^1^Department of Rehabilitation, The Affiliated Hospital of Southwest Medical University, Luzhou, China; ^2^Department of Rehabilitation Medicine, Southwest Medical University, Luzhou, China; ^3^Rehabilitation Medicine and Engineering Key Laboratory of Luzhou, Luzhou, China; ^4^Department of Clinical Medicine, Southwest Medical University, Luzhou, China; ^5^School of Clinical Medicine, Guangxi Medical University, Nanning, Guangxi, China

**Keywords:** rehabilitation, bibliometric analysis, commentary, post-stroke, dysphagia

We would like to share some ideas on the article “*Research trends and hotspots of post-stroke dysphagia rehabilitation: a bibliometric study and visualization analysis”* (He et al., 2023).

The study aimed to provide a comprehensive understanding of the current state of research on post-stroke dysphagia rehabilitation from its inception until July 27, 2023 through bibliometric analysis. The core collection of the Web of Science (WoS) database was searched. The data retrieval strategy is as follows: TS = (stroke OR apoplexy OR “cerebrovascular accident” OR “brain vascular accident” OR “cerebral hemorrhage” OR encephalorrhagia OR “cerebral ischemia”) AND TS = (“Deglutition Disorder” OR “Swallowing Disorder^*^” OR Dysphagia OR “Oropharyngeal Dysphagia” OR “Esophageal Dysphagia”) AND TS = (rehabilitation OR recovery). And 1,097 documents related to post-stroke dysphagia rehabilitation were analyzed in detail by bibliometric analysis using VOSviewer, CiteSpace, and Bibliometrix R software packages. The study found that the United States had the most publications (215 articles) and was the most influential country. “Dysphagia” was the journal with most publications (100 articles) and the most citations (4,606 citations). Hot topics focus on PSD-related guidelines, the accuracy, and validity of screening tools, non-invasive brain stimulation, transcranial magnetic stimulation (rTMS), tDCS, and acupuncture for PSD rehabilitation.

We would like to make four points about this study. First, the bibliometric analysis of the study was very standardized with search terms. However, the search terms may be relatively incomplete, which may affect the search results. Our updated search strategy is as follows: TS = (stroke OR apoplexy OR “cerebrovascular accident” OR “brain vascular accident” OR “cerebral hemorrhage” OR encephalorrhagia OR “cerebral ischemia” OR “cerebral infarction”) AND TS = (“Deglutition Disorder” OR “Swallowing Disorder^*^” OR Dysphagia OR “Oropharyngeal Dysphagia” OR “Esophageal Dysphagia”) AND TS = (Rehabilitation OR Recovery OR “Physical therap^*^” OR “Exercise therap^*^”). The rest settings were as in the original text (He et al., 2023). We retrieved 1,318 related articles, including 1,077 articles and 241 reviews. The analytical tools include citespace, VOSviewer, and Bibliometrix. CiteSpace visualizes the dynamics of the bibliometric network over time, and VOSviewer is mainly used to visualize the bibliometric network. Bibliometrix is an R package that contains a range of functions for quantitative scientometric research (Sun et al., 2022). The study analyzed the current research status and hotspots of post-stroke dysphagia rehabilitation in detail by using the main bibliometric analysis tools.

Second, considering the comprehensive information about the full text and citation analysis, the authors only selected literature from the WoSCC database. Although WoS is the most frequently used database, searching the literature using only a single database may have caused bias and limited the comprehensiveness of the analysis. We note that some bibliometric studies chose other databases such as Scopus, PubMed, and Embase. It is difficult to integrate and analyze literature from different databases. However, with an understanding of the characteristics of the literature on stroke dysphagia rehabilitation included in each of the different databases, it is easier for the reader to understand the representativeness of this section of the studies analyzed by the authors. Bibliometric analyses focus on the current state of research, hotspots and frontiers of knowledge, but they may not provide a detailed analysis of the content and quality of the studies. For a more comprehensive understanding of stroke dysphagia rehabilitation research, it is advisable to combine bibliometric analyses with a detailed qualitative analysis of the included studies. We noted that some researchers have analyzed and provided in-depth reviews of bibliometric findings, which can help readers gain a detailed understanding of the field (Zheng et al., 2022; Sang et al., 2023; Tang et al., 2023). The authors of the original article analyzed the pathophysiology and neuroplastic processes of PSD, comorbidities, swallowing screening and assessment methods, and swallowing rehabilitation methods in the discussion section. Rehabilitation of PSD was the subject of the original article. Although the bibliometric results only suggest that non-invasive brain stimulation and acupuncture are hot topics, we note that botulinum toxin injection therapy, electrical stimulation, and balloon dilatation have had a steady scientific output in recent years and are widely used in clinical practice (Chandrasekhara et al., 2017; Xie et al., 2022; Wang et al., 2023). Therefore, it is suggested that the authors' discussion section should provide a more in-depth review about these rehabilitation methods.

Third, many indicators are used to quantitative analysis the productivity and influence of scientific research, such as the publication number, influence factor (IF) of journals, citation, and h-index. However, each indicator has its limitations. Therefore, many bibliometrics studies tend to use a variety of indicators to analyze from different angles, so as to obtain a more comprehensive and objective evaluation. For the impact indicators of countries and institutions, only the total number of citations is listed (Table 1) and the citation situation for authors is not analyzed (Table 3). Citation analysis may be more important in bibliometric analysis, while the total number of citations is related to the total number of papers published and the time of publication. Only analyzing the total number of citations may not be comprehensive enough, and it is suggested that the authors can add average number of citations and the mean density of citations. Besides, the study lists the h-index for the countries and authors with the highest number of publications. It may not be sufficient to use the h-index to indicate the performance of countries and authors alone. h-index may lag behind the ranking of emerging researchers in the field. The m-index is a variant of the h-index, obtained by dividing the h-index by the number of years since an individual's first publication. m-index reduces the time bias inherent in the h-index and can be used to compare the performance of researchers at different stages of their careers (Ha et al., 2021). The g index is defined as an author's top g articles that have been cited an average of g times or at least g^2^ times. It considers more of the performance of well-cited articles while keeping all the good properties of h-index, which can better represent overall achievements than h-index (Egghe, 2006). A bibliometric study on robotic surgery quantifies the quality and quantity of author productivity by integrating the h-index, g-index, and m-index of authorship (Musbahi et al., 2022). Lin et al. (2022) used the h-index and g-index to measure country and journal impact in a bibliometric analysis study of autophagy in lung disease. Xiao et al. (2022) also used the h-index and g-index in their bibliometric analysis study to assess in detail the impact of countries, authors, institutions and journals. Therefore, it is desirable to use more indicators to synthesize the impact of researchers.

Finally, the discussion section of this study provides a systematic review of research topics and frontiers, including pathophysiology and neuroplasticity of PSD, comorbidities, swallowing screening and assessment methods, and models of swallowing rehabilitation, so that researchers interested in this field can have an updated understanding of PSD rehabilitation. In particular, through the bibliometrics method, the authors found that the research related to rehabilitation of swallowing disorder after stroke also involves psychology, education and social science, which may not have been paid enough attention in the past. This is different from other bibliometric studies and is worthwhile for those interested in bibliometric analysis.

## Author contributions

MH: Writing—original draft. CZ: Writing—original draft. ZL: Writing—original draft. JW: Writing—review & editing.
